# It’s only human

**DOI:** 10.1186/gb4159

**Published:** 2014-02-12

**Authors:** Neil Hall

**Affiliations:** 1 Centre for Genomic Research, University of Liverpool, Liverpool L69 7BZ, UK

## 

In the field of genomics we academics have always worked closely with our industrial partners who develop the tools of the trade. Recently this relationship has been dominated by Illumina, a company who, more than any other, has propelled DNA sequencing from a glamour branch of genetics to the most exciting area of biomedical science to work in. For the last few years Illumina has left most of us in besotted awe of their machines; like wide-eyed Apple groupies we never tire of discussing the awesomeness of their technology. Illumina have powered the genomic revolution and we can only hold up our hands in admiration, as we have been beneficiaries of their success just as much as their shareholders. You may almost call it symbiotic - but you would be wrong. Recent events should remind us that we are customers not colleagues.

On January 14 Illumina made a major announcement at the JP Morgan Healthcare conference [1]. They unveiled the Hiseq X Ten, an amazing new instrument that they say:

● Can sequence a human genome for less than $1,000 (so we can all stop predicting when that’s going to happen now)

● Can only be bought in batches of 10 at an eye watering $10Million

● Can only be used to sequence human DNA

Yes! it can ONLY be used to sequence human DNA!

Heckles should always be raised whenever a scientist hears something from a science-led company that sounds like bad science. Now, last time I checked, and it has been a while since I read much on basic DNA chemistry, human DNA is … well… deoxyribonucleic acid; in a similar way to mouse DNA, banana DNA, armadillo DNA and *Mycobacterium tuberculosis* DNA. Many of my research hypotheses are based on this naïve assumption, but when I heard about the X Ten, it seemed Illumina had hit upon some unique intrinsic quality of our blessed DNA that makes it particularly… erm, humany, allowing their new machine to only work on our highly specialized genetic code. The intelligent design army would be delighted, and that’s always a bad sign. But people who have been hanging around the next-generation sequencing scene in the last five years or so were crumpling their noses at this news as everything about it smelled fishy, not just the special properties of human DNA bit. Why not announce it at the AGBT meeting like everyone else? Why 10 at a time? (Can you imagine the iPad Ten or the Ferrari Ten?) What were Illumina up to here?

First, let’s look at why this machine only sequences human DNA - it’s because the conditions of purchase require that you only use it for human DNA. It is not optimized for human DNA, Illumina just don’t want you to use it for anything else. In the same way that a Hewlett Packard printer *could* work with third party print cartridges, or DVDs bought in Europe *could* work in the USA; the reason they don’t is that technical or legal roadblocks have been built to limit these systems. Not because it makes them better, it just keeps the customers under control. And if you think that this restriction will be like the days when we were only supposed to use Taq that was ‘licensed to PCR’ but we all used the cheap stuff from Promega anyway, it won’t be - this rule will be enforced.

The motivation behind all this is revealed in *where* the X Ten was announced; at a healthcare investment conference. It’s clear who the perceived beneficiaries of this new machine will be. Also of note is Illumina’s hand-break U-turn in strategy from ‘cheap sequencing for all’ to ‘cheap sequencers for the wealthy centers’ by only selling the machine in packs of 10*.* Although instruments were announced that were aimed at smaller labs, the NextSeq and a cheaper MiSeq, these can’t compete in cost per base with the X Ten. Blogger Mick Watson, usually a stalwart, pom-pom spinning Illumina cheerleader (sorry Mick, it’s true), has dubbed this ‘de-democratizing sequencing’ [2]. The reason for this move is not clear to me but I expect it helps in policing the only-human contract - production centers are far less likely to perform homebrew experiments.

All of this is aimed at engineering market separation between human genome sequence production and everyone else. This is worrying in part because the bioinformatics and laboratory developments at these centers have driven research in all areas of genomic science. If they are working on a different technology to the rest of us we all suffer.

So… why would Illumina wish to segregate the genomics community? My theory is as follows.

In the last year or so BGI bought Complete Genomics [3] and has been competing quite aggressively on price with Illlumina for large-scale human DNA sequencing. Currently the business model of Complete Genomics has been only to focus on human sequencing, as they don’t sell technology, they sell a service. Illumina need to bring out new, cheaper technology to ensure their market dominance in the all-important human sequencing arena but they can see little advantage in making it cheap for everyone else, as it will just reduce their bottom line. Hence a super-sequencer that is locked to human seems like a pretty neat idea to the accountants.

I call this the ‘Accountants sacrificing humans for profit’ theory. Amongst those I have asked, my theory has been widely accepted. On the acceptance spectrum, it is pretty close to the theory of evolution and way ahead of string theory. Also, I may not be the first person to propose this theory [4].

This sort of control is akin to region encoding of DVD players, it is a technology which serves to allow industry greater control over the market, but makes the product less useful. In science, I can’t think of a parallel. I wonder if the genomics community has found themselves in a unique situation where they are subject to the whims of a commercial cartel. Are there companies that make telescopes that can only be used to study spiral arm galaxies and not nebula ones? Or mass spectrometers that will only work with carbon containing compounds? Yet it seems that some institutes are quite willing to be complicit in this exercise and have signed up to have their hands tied in return for cheap sequence. So that as the thousands of genomes roll off the new X Ten centers, Illumina sequence will remain as the industry standard for human genomics, which places them in prime position for rare disease testing, patient stratification and so on, while ensuring the rest of the bioscience community does not slipstream any benefit from the technology.

I am sure neo-capitalist readers are gasping to tell me how market economics works, but I DO GET IT. The reason Illumina have invested so many research dollars in this technology is to cash in on the clinical market, that’s how these companies work. But we academics have been leveraging the perceived potential of personal genomics, and the tools developed for it, to drive forward research and discovery in all areas of biological science. We have imposed the power of next-generation sequencing on areas of research such as food security, global climate change and biodiversity, but apparently this technology was never meant for everyone - it was only really developed for the clinical market. I imagine that some may say that us freeloaders were never going to pay for the ride so we deserved to be thrown off the train - that’s market economics - *live with it*.

But how far do we take this argument? Is it OK that you don’t get to use the best and cheapest technology if you are sequencing pathogens that cause HIV, tuberculosis or malaria, or if you are developing drought tolerant crops or cataloging endangered species? If the technology exists to drive this research forward, is it OK to prevent people from using it? When did we start leaving these decisions to industry? I am somewhat surprised that there has been so little discussion around the principle of a company dictating to scientists what samples are allowed to be run on their products.

One of the things about working in academic science that gives me that warm fuzzy feeling inside is that we are a community held together by a passion for discovery. Regardless of if we work on cancer, infection, plant development or intertidal bivalve diversity, whether we are tenured professors or PhD students, we have a common respect for our shared discipline of science. There is a humility and nobility to our existence that gives us an inner smugness, like the Type 1 Volkswagen Beetle owners who wave at each other as they pass on the road. Maybe the problem I have with Illumina’s strategy is that it has nothing to do with discovery, science or even human health. It grates like fingers scraping the chalkboard of my academic utopia. This policy, which could prevent researchers from using technology that may help to solve some of the great challenges affecting humankind, is motivated by the same thing that caused some pharmaceutical companies to try to block generic drug licenses for developing countries. As the greatest market manipulator of them all, Bill Gates, once said, ‘You know capitalism is this wonderful thing that motivates people, it causes wonderful inventions to be done. But in this area of diseases of the world at large, it’s really let us down’ [5].

## Competing interests

Neill Hall’s lab has purchased a lot of Illumina products and this is unlikely to change in the foreseeable future.
